# Selection and Surface Modifications of Current Collectors for Anode-Free Polymer-Based Solid-State Batteries

**DOI:** 10.3389/fchem.2022.934365

**Published:** 2022-07-07

**Authors:** Oihane Garcia-Calvo, Antonio Gutiérrez-Pardo, Izaskun Combarro, Ander Orue, Pedro Lopez-Aranguren, Idoia Urdampilleta, Andriy Kvasha

**Affiliations:** ^1^ CIDETEC, Basque Research and Technology Alliance (BRTA), San Sebastian, Spain; ^2^ Centre for Cooperative Research on Alternative Energies (CIC EnergiGUNE), Basque Research and Technology Alliance (BRTA), Alava Technology Park, Vitoria-Gasteiz, Spain

**Keywords:** anode-free batteries, solid-state batteries, solid electrolyte, current collector, lithium metal

## Abstract

Anode-free batteries (AFB) have attracted increasing interest in recent times because they allow the elimination of the conventional anode from the cell, exploiting lithium inventory from a lithiated cathode. This implies a much simpler, cost-effective, and sustainable approach. The AFB configuration with liquid electrolytes is being explored widely in research but rarely using solid electrolytes. One of the main issues of AFB is the poor reversibility of the lithium-plating/striping process at the anode side. Therefore, in this work, different metal foils have been tested as anode current collectors (CC), and copper foil has been selected as the most promising one. Surface modifications of the selected copper foil have been achieved by its coating using composite layers made of carbon and different metal nanoparticles—such as Ag, Sn, or Zn—in different proportions and with different amounts of a binder. The impact of such coatings and their thickness on the electrochemical performance of single-layer solid-state anode-free pouch cells, based on a PEO electrolyte and a LiFePO_4_ cathode has been systematically studied. Consequently, a post-mortem analysis of the investigated solid-state AFB is also presented, trying to identify and elucidate possible failure mechanisms to enhance the electrochemical performance of solid-state AFB in the future.

## 1 Introduction

Modern society and economy decarbonization strongly require the development of long-lasting, highly functional, safe and cheap batteries. Solid-state batteries (SSB) are one of the most promising types of advanced batteries because of many such advantages as: 1) enabling the use of high energy electrode materials towards increasing energy density up to 500 W h·kg^−1^, 2) intrinsic thermal stability and non-flammability of solid electrolyte materials for safety improvement, and 3) stable “solid electrolyte/electrode” interfaces causing enhanced durability. (Ahniyaz et al., 2021; Thieu et al., 2021). However, the cost and sustainability of solid-state batteries seem to be one of the main weak points of this disruptive technology. Many approaches have been proposed to reduce the potential cost of SSB: 1) using the equipment of conventional lithium-ion battery plants, (Duffner et al., 2021); 2) deployment of roll-to-roll methods to manufacture solid electrolytes and electrodes, (Kim et al., 2020), 3) *in-situ* solidification techniques, (Albertus et al., 2021); (iv) anode-free batteries (AFB), (Xie et al., 2020; Tong et al., 2021), etc., In this connection, it seems that the use of so-called anode free batteries, which do not require the employment of extremely reactive and expensive 20–100 μm thick Li metal foils, is a reliable approach to significantly reduce the cost. In addition, the gain in volumetric energy density can be especially large compared to Li metal SSB with a high negative to positive areal capacity (N/P) ratio, due to the high specific volume of lithium. The elimination of Li metal during cell manufacturing simplifies assembly process and helps to reduce the energy demands during the production. (Heubner et al., 2021).

The main difference between anode-free SSB and lithium metal SSB is the concept of the negative electrode. In more detail, during an anode free cell charge, lithium ions deintercalated from a lithiated cathode material deposit in Li metal form -on a current collector or another host. In turn, while the anode free cell is discharging, deposited Li metal is stripping out from the current collector. Thus, AFB exploits a limited Li inventory from the cathode and, therefore, the typical failure mode of AFB is continuous loss of cyclable lithium throughout the entire cycling (Niu et al., 2021). This loss is usually caused by poor reversibility of the Li-alloying and Li-stripping/plating processes, the formation of electrochemically inactive Li deposits, detrimental reactions with an electrolyte, etc. In this connection, the reversibility of the lithium stripping/plating process must be higher than 99.99% to maintain Li inventory almost constant and, in this way, ensure the long-term cyclability of AFB. Therefore, this is one of the main foci of recently published reports on anode-free batteries, where several approaches have been suggested to improve the efficiency and reversibility of the Li stripping-plating process. The main proposed approaches are the following: 1) optimization at the cell level (formation process, pressure, testing protocol, etc.), 2) improvement of electrolyte formulation, and 3) optimization of the current collector, including surface treatments and coatings. Consequently, ion conducting interlayers or applied stack pressures can be highly beneficial towards obtaining a homogeneous Li plating in anode-free cells and will help to achieve industrial requirements (Krauskopf et al., 2020).

Dahn’s group (Louli et al., 2021) reported that the optimization of an anode free cell formation conditions, applied pressure, and testing protocols can significantly improve capacity retention and Coulombic efficiency (CE). Louli et al. (2019) found that the increase of initial pressure from 75 kPa to 2200 kPa benefits the cycling performance and Coulombic efficiency of anode-free cells due to the formation of more compact Li metal deposits. Genovese et al. (2019) reported a synergistic effect of “hot formation” at 40°C and the application of 1200 kPa pressure on the electrochemical behavior of the studied AFB. The observed positive effect of cell formation at 40°C was partially caused by an increased CO_2_ gas generation which could act as a beneficial additive. The same group recently reported that asymmetric testing protocols with a charge C-rate lower than the discharge are optimal for minimizing lithium inventory loss during AFB operation (Louli et al., 2021). In addition, a specialized intermittent high energy cycling protocol, with mixed low depth of discharge cycles and interspersed high energy deep discharge cycles, has been developed towards a compromise between extending lifetime and the ability to provide high energy density occasionally.

Another way to improve the AFB performance is related to the development of advanced electrolytes that favor the formation of a stable SEI layer to reduce side reactions, which allows the creation of a more stable interface to achieve a CE close to 100% (Eldesoky et al., 2021; Niu et al., 2021). For example, Qian et al. (2016) demonstrated that the use of a highly concentrated liquid electrolyte (4M LiFSI-DME) strongly improves the electrochemical performance of an anode-free Cu/LiFePO_4_ battery due to the formation of a SEI layer with a significant amount of Li-containing inorganic compounds, which possesses enhanced ionic conductivity and mechanical stability. As another approach, Zegeye et al. (2020) laminated a LLZTO/PEO composite electrolyte on both the cathode and Cu foil surfaces with an ultrathin thickness of 7–10 μm by the spin-coating method, an innovative strategy which can inspire the design and development of compatible electrolytes for AFB.

Nevertheless, one of the most promising and effective ways to improve the overall reversibility of the Li stripping/plating process is the optimization of the negative electrode of AFB. For example, Menkin et al. (2021) demonstrated that the current collector choice and its pre-treatment are essential for the realization of practical AFB. Another approach is the use of three-dimensional current collectors with developed surface area, which effectively decreases real current density and, in this way, enhances the reversibility of the Li metal plating/stripping process, avoiding Li dendrite plating (Tong et al., 2021). In this sense, Yi et al. (2019) studied the lithium nucleation kinetics in laser-induced graphene on Cu foils and explored the potential of using laser processing for large-scale fabrication of high-performance current collectors to stabilize the metallic anode (Yi et al., 2019). On the other hand, surface modification of current collectors with different elements (e.g. Sn, Ag, Au, Ge, and C) effectively helps to improve the electrochemical performance of AFB. To date, the most impressive result on surface modification of current collectors for AFB was reported by Lee et al. (2020) on the development of an AF-SSB based on a sulfide solid electrolyte, lithium nickel manganese cobalt oxide based (LiNiMnCoO_2_) cathode, and disruptive Ag/C composite based anode that effectively enables the developed AF-SSB. The elaborated composite anode was based on silver nanoparticles, carbon black, polyvinylidene fluoride (PVdF) binder, and a stainless steel foil substrate. The anode with a weight ratio of Ag/C of 1:3 demonstrated outstanding Li plating/striping reversibility, reaching a CE of >99.99% in the solid-state cell with a positive electrode having a loading of 6.8 mAh·cm^−2^. As a result, the developed AF-SSB in pouch format has reached about 1000 cycles with an average CE of >99.8%.

Inspired by previous studies, herein we report the results of a systematic study regarding the selection and optimization of a lithophilic current collector for anode free solid-state batteries with a LiFePO_4_-based cathode and a solid composite electrolyte based on the PEO-LiTFSI system. We demonstrated that the coating of a copper foil current collector by a mixture of carbon black and different metal nanoparticles significantly improves the reversibility of the lithium metal stripping/plating process and, in this way, enhances the electrochemical performance of an anode-free solid-state battery in pouch format.

## 2 Experimental Section

### 2.1 Benchmarking of Anode Current Collectors

Among the possible commercial alternatives, five different current collectors were tested. Table 1 summarizes commercial current collectors tested in anode-free SSB, before and after chemical etching or coating.

**TABLE 1:** Selection of commercial current collectors.

Material	Reference	Thickness (µm)	Provider
Cu	NC-WS	8	Furukawa
Cu	SE-Cu	10	Schlenk
Ni	Nickelband	11	Schlenk
CuNi_3_Si	Copper alloy foil	10	Schlenk
Cu/C	Double carbon-coated Cu foil	11 (9 + 2)	Xiamen Tmax

### 2.2 Surface Modification of Anode Current Collectors

#### 2.2.1 Chemical Etching

The passivation layer on the investigated current collectors was removed by washing with dilute hydrochloric acid (HCl 37%, Fisher Scientific) for ∼1 min and subsequent rinsing with deionized water and acetone (99.8%, Acros). The current collectors were finally dried at room temperature with argon gas and stored under vacuum in a pouch bag until pouch cell assembly to avoid further surface oxidation.

#### 2.2.2 Preparation of Nanocomposite Layers

Commercial nanoparticles (NPs: Ag, 50–80 nm; Zn, 60–75 nm; Sn, 60–80 nm, all from United States Research Nanomaterials) and carbon black powder (C45, Imerys Carbon & Graphite) were selected as the anode materials. NPs and C45 were mixed in an optimized weight ratio in N-methylpyrrolidone (NMP, Merck), which containing among 10–20 wt% of polyvinylidene fluoride (PVdF, Solef^®^ 5130, Solvay). NMP was slowly added to the mixture under constant stirring using a mechanical mixer (RW 20 digital, IKA) to prepare the anode slurry. The slurry was then coated on the current collector by doctor blade casting (200 μm gap, 90 mm s^−1^ speed), and thin deposits were dried in a convection oven at 80°C for 30 min. The obtained negative electrode was calendared in order to reduce the porosity, and was then dried under vacuum at 100°C for 12 h. Table 2 summarizes the main characteristics of the developed composite coatings.

**TABLE 2:** List of different coatings on the copper foil current collector (Furukawa).

#	NPs/C (weight ratio)	PVdF (wt%)	Coating thickness (µm)
C_01	Ag, 1:3	20	9 ± 1
C_02	Ag, 1:2	20	16 ± 2
C_03	Ag, 1:1	20	12 ± 2
C_04	Ag, 1:3	15	14 ± 1
C_05	Ag, 1:3	10	15 ± 1
C_06	Zn, 1:3	10	17 ± 1
C_07	Sn, 1:3	10	8 ± 1

### 2.3 Cathode and Solid Electrolyte Fabrication for Anode-Less Pouch Cells

For safety, toxicity, and cost-effective reasons, LiFePO_4_ (LFP) has been selected as the cathode active material for anode-free prototypes, because it possesses a gravimetric capacity similar to other metal transition oxides and an electrochemical window compatible with solid polymer electrolytes based on polyethylene oxide (PEO). Moreover, it is a reference cathode material since it presents a flat and stable voltage plateau at around 3.4 V vs. Li/Li^+^, which facilitates the identification of the polarization during the charge/discharge steps of a cell based on the Li/LFP chemistry. (Gutiérrez-Pardo et al., 2021).

Hence, LFP-based cathodes developed for this work contain carbon-coated Al as the cathode current collector (thickness 22 µm), and the active layer containing commercial carbon coated lithium iron phosphate (LiFePO_4_) powder (D_50_: 2–4 µm) as active material, conductive additive C-ENERGY Super C45 carbon black (IMERYS Carbon & Graphite) and PEO (Mn 4.10^5^ g mol^−1^)-LiTFSI (EO/Li∼20) as catholyte in a 75/5/20 wt% respectively, with a loading of active material between 0.54 and 0.65 mAh·cm^−2^ and a density of about 2.0 g cm^−3^. This cathode formulation was successfully upscaled to a 180 g batch to manufacture positive electrodes at pilot plant scale. An up-scaled positive electrode was effectively validated by comparison of morphology and electrochemical response in solid-state coin cells. The preparation of the LiFePO_4_ composite cathode is detailed in our previous report. (Thieu et al., 2021).

Since 1980s (Armand, 1983), PEO has been representing as one of the most employed polymers in the SSB technology, due to its high solvating ability for lithium salt, good processability and low cost. PEO’s low glass transition temperature (T_g_, -60°C) allows the polymer chains to transport mobile Li^+^ ions. However, being a semi-crystalline polymer, ion conduction is only possible in the molten state, where PEO is a viscous liquid with poor mechanical properties and the solid matrix can no longer act as a barrier against Li dendrite growth (Xue et al., 2015). The most common strategy to improve the mechanical and electrochemical properties of PEO-based electrolytes, as well as decrease the melting temperature and the degree of crystallinity of the PEO matrix, is the addition of inorganic nanoparticles, such as Al_2_O_3_ (Lago et al., 2015). In this sense, and based on our previous experience, the solid electrolyte was prepared by dispersing 10 wt% of Al_2_O_3_ nanoparticles (5 nm, United States Research Nanomaterials) in an acetonitrile solution of PEO (Mn 6.10^5^ g mol^−1^)-LiTFSI (EO/Li∼20) (12 wt%). The slurry preparation for the up-scaled solid electrolyte was carried out in the dry room (dew point below -50°C). Thus, the electrolyte membrane was prepared by solvent casting over a Teflon sheet employing a doctor blade (1200–1400 μm gap, 120 mm s^−1^ speed). The casted solid electrolyte membranes were allowed to evaporate at 35°C for 2 h in a convection oven before being dried under reduced pressure for 18 h at 60°C. Solid electrolyte membranes were finally detached from Teflon sheets and stored in the dry room before usage. Homogeneous solid electrolyte membranes with a surface area of more than 350 cm^2^ and an average thickness of 55–75 μm (depending on gap) were obtained for further assembly of solid-state cells.

### 2.4 Characterization Methods

The surface morphologies of bare current collectors and different coatings were analyzed by Field Emission-Scanning Electron Microscope (FE-SEM, Zeiss Ultra Plus), operated at a voltage of 5 kV using secondary electrons mode.

All-solid-state single-layer pouch cells (50 × 60 mm^2^), with a capacity of ∼30 mAh, were manually assembled by stacking of bare or modified current collector or 50 μm thick Li metal foil (Albemarle) as anode, the up-scaled solid electrolyte, and a composite LFP cathode (LL 0.50–0.65 mAh·cm^−2^, D 2.0 g cm^−3^). Supplementary Figure S1 depicts the typical assembly procedure of the single-layer anode-free SSB prototype employed in this study. Finally, the assembled solid state stack was placed in a pouch bag and sealed under vacuum. All the operations during the battery assembly process were performed under dry room conditions with a dew point below -50°C (<24 ppm of water). Before cell cycling, an external pressure with an initial torque of 1 N m (∼600 kPa) was applied to a freshly assembled SSB, which was placed between two 6 mm-thick stainless-steel plates (11 x 14 cm^2^). Such a constant volume type setup was used for better control of the initial pressure magnitude and to improve the contact among all cell components. Two identical cells were assembled for each variation to ensure reproducibility, and one of them is reported in the results.

Once assembled, pouch cells were kept at 60°C for 3 h to further improve contacts at the “anode/solid electrolyte” and “cathode/solid electrolyte” interfaces, followed by galvanostatic cycling at a charge–discharge current rate of 0.1C within a voltage window of 2.0–3.8 V at 60°C, using the BaSyTec cell test system. In parallel, for post-mortem analysis (PMA) purposes, 2 cells were cycled under similar conditions (“AF_02” and “AF_04”), and 2 cells (“AF_01” and “AF_03”) were just kept at 60°C for 3 h and charged at 0.1C up to 3.8 V before PMA, to establish them as a baseline for further comparisons. Table 3 summarizes the main characteristics of the investigated cells.

**TABLE 3:** Post-mortem characterization of pouch cells.

#	Anode (µm)	Cathode (LL, mAh·cm^−2^)	Solid electrolyte (µm)	Conditions before post-mortem analysis
AF_01	Washed Cu foil (8)	0.55	70	60°C, 16 h
AF_02	Washed Cu foil (8)	0.65	68	i) 60°C, 16 h; ii) 0.1C/0.1C, 2.0–3.8 V at 60°C
AF_03	C_05 (15)	0.54	68	60°C, 16 h
AF_04	C_05 (15)	0.57	77	i) 60°C, 16 h; ii) 0.1C/0.1C, 2.0–3.8 V at 60°C

For the post-mortem analysis, the pouch cells were carefully disassembled under an Ar-filled glovebox, and an air-tight transfer tool was used to transfer air-sensitive samples directly from the Ar-filled glovebox to the vacuum chamber to the SEM equipment. The morphological characterization of these pouch cells was conducted via scanning electron microscopy (SEM) in a FEI Quanta 200F SEM-EDX station at 20 keV of acceleration voltage, using either secondary (ETD) or backscattered (BSED) electrons. Additionally, the ion-milling of the cross-section of fresh and cycled pouch cells was carried out with Hitachi IM4000PLus equipment. The cell was ion beam milled at 90° for 3 h and 1 h at an acceleration voltage of 6 keV and 1 keV, respectively, operating in cryostat mode at -70°C to avoid the melting of the investigated solid electrolyte based on PEO polymer.

## 3 Results and Discussion

### 3.1 Selection of Anode Current Collectors

**FIGURE 1:**
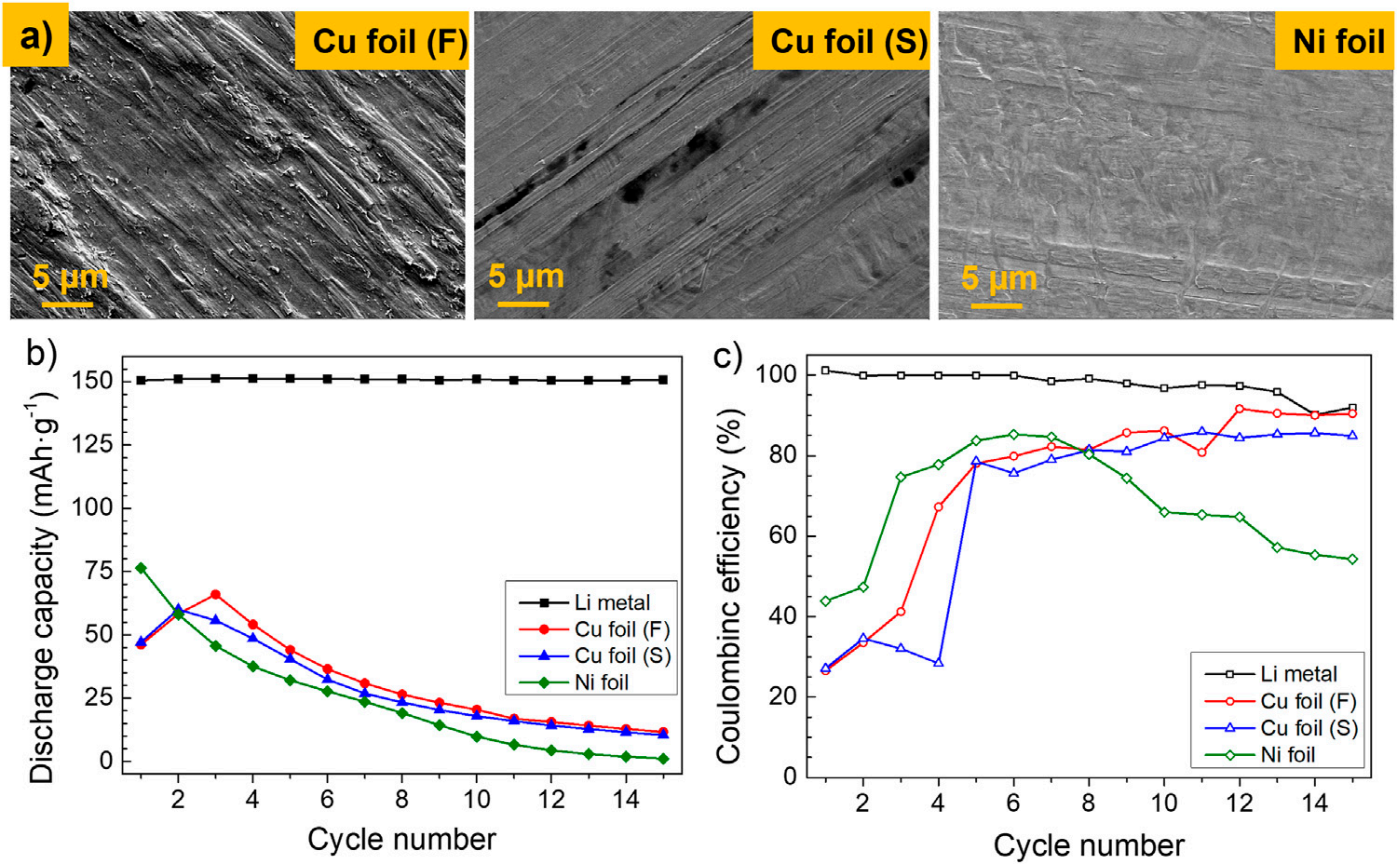
**(A)** FE-SEM micrographs of different investigated anode current collectors, **(B)** discharge capacity, and **(C)** Coulombic efficiency of single-layer pouch cells using such anode current collectors. Cycling conditions: 60°C, 1 N m, DoD 100%, 2.5–3.8 V, 0.1C–0.1C, and charge cut off current 0.05C.

A benchmarking of different materials has been performed in order to select the most promising anode current collector (CC), considering chemical and electrochemical factors. Figure 1 shows FE-SEM pictures of three different metal foils as commercial CC: Cu foil, from two different providers (Furukawa and Schlenk), and Ni foil (Schlenk), together with their electrochemical behavior in single layer pouch cells. Ni foil presents a smooth and homogeneous surface with no pores along its surface, while Cu from Furukawa exhibits some roughness, although the surface is uniform, and pores are not seen. The copper foil supplied by Schlenk exhibits a dissimilar surface, lacking overall homogeneity. Galvanostatic cycling of pouch cells containing such bare current collectors exhibits discharge capacities much lower than values obtained using conventional Li metal foil as anode, and a capacity that fades with the number of cycles in all cases (Figure 1B). Such cells present a low Coulombic efficiency at the beginning, reaching values of about 80%, which is maintained in the case of both Cu foils, but decreases progressively in case of Ni foil (Figure 1C). In each cell using two different copper foils, low initial discharge capacities and, therefore, low Coulombic efficiency values are seen, probably due to an unstable SEI layer and “dead” lithium formation. On the contrary, a high stable cycling with a discharge capacity of 150 mAh·g^−1^ and a CE above 95% during the first 15 cycles is shown in the lithium metal solid-state cell.

In parallel, alternative commercially available foil current collectors have also been studied and tested: an alloy containing Cu, Ni, and Si (CuNi_3_Si) and a carbon-coated copper foil (Cu/C). Supplementary Figure S2 shows the FE-SEM micrographs of the surface of such foils and the cycling of cells which contain them as anode current collectors. Concerning their microstructure, CuNi_3_Si shows a homogeneous and flat surface, with some micropores throughout the surface. Cu/C foil exhibits a rough but uniform surface coating. Regarding the galvanostatic cycling of cells (Suppl. Figure 2B), the first cycle in both cases presents a discharge capacity slightly higher than the cells using the Cu or Ni metal foils. Nevertheless, the initial discharge capacity values decrease strongly, and the Coulombic efficiency is low and unstable in both cases.

**FIGURE 2:**
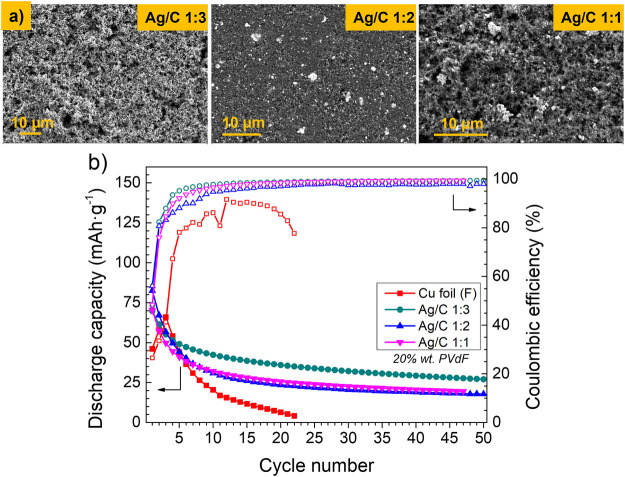
**(A)** FE-SEM micrographs of Cu foil and Ag/C coatings with different Ag:C ratios on Cu foil as the anode current collector. **(B)** Discharge capacity and Coulombic efficiency of single-layer pouch cells using anode current collectors coated by different Ag/C nanocomposite layers. Cycling conditions: 60°C, 1 N m, DoD 100%, 2.5–3.8 V, 0.1C–0.1C, and charge cut off current 0.05C.

Considering these observations, Cu foil supplied by Furukawa has been selected as the reference anode current collector because AF_SSB based on this CC presents the highest discharge capacity, a moderate capacity fading in comparison to the other CC, and a Coulombic efficiency that increases with the cycle number and tends to stabilize above 80% after the first five cycles. Different coatings and/or treatments on the surface of reference CC have been carried out to improve reversibility of the process and, in this way, increase both the discharge capacity and Coulombic efficiency of the AF cells, trying to solve the instabilities observed at the beginning of cycling of the cells based on the bare CC.

### 3.2 Modification of the Reference Current Collector

First, the reference Cu foil has been washed with HCl. Supplementary Figure S3 shows the different color of the Cu current collector before and after chemical etching, which confirms oxide layer removal after such treatment, as it has been previously reported by others (Menkin et al., 2021). Cell cycling using washed copper foil presents a higher discharge capacity, with a fall when increasing the cycle number, similar to the bare copper foil. Moreover, the discharge capacity is higher in the first two cycles, which suggests a higher lithiophility and lower interfacial resistance, and the Coulombic efficiency tends to stabilize after the second cycle. However, the combination of such initial etching with a subsequent coating of the CC has presented noticeable reactivity issues (corrosion). For that reason, this chemical treatment has been discarded for further coating developments in this study.

Afterwards, different nanocomposite layers have been deposited on the surface of the untreated reference Cu foil current collector, varying some parameters in the composition to study the impact on the electrochemical behavior of the anode free cells. First, Ag nanoparticles were mixed with carbon black in different proportions, maintaining a fixed amount of 20 wt% of the PVdF binder in the composition. Figure 2 shows the FE-SEM micrographs of different coatings as well as the electrochemical cycling of cells containing these coatings on the CC. Irrespective of the Ag:C ratio, all the coatings exhibit a uniform and homogeneous appearance and improve the electrochemical properties of the cells: an initial discharge capacity of 70–80 mAh·g^−1^, followed by a strong capacity decay for five cycles. From the fifth cycle, the discharge capacity moderates its fall, maintaining a slight fading in contrast with the cell using the bare Cu foil, which shows a strong capacity fading and fails completely after 22 cycles. Regarding the CE, the cells containing Ag:C coatings exhibit a low initial value which tends to stabilize close to 100% after five cycles, maintaining such values for the following 50 cycles. In turn, without CC coating, the cell reaches a maximum CE of 90%, which falls quickly after a few cycles. The cell containing the coating with an Ag:C 1:3 ratio on the reference CC exhibits better capacity retention, and the CE reaches values close to 100% before cells with other Ag:C proportions. For that, different layers containing metal nanoparticles and carbon black powder have been studied afterwards, varying different parameters but keeping 1:3 ratio in weight between such components.

**FIGURE 3:**
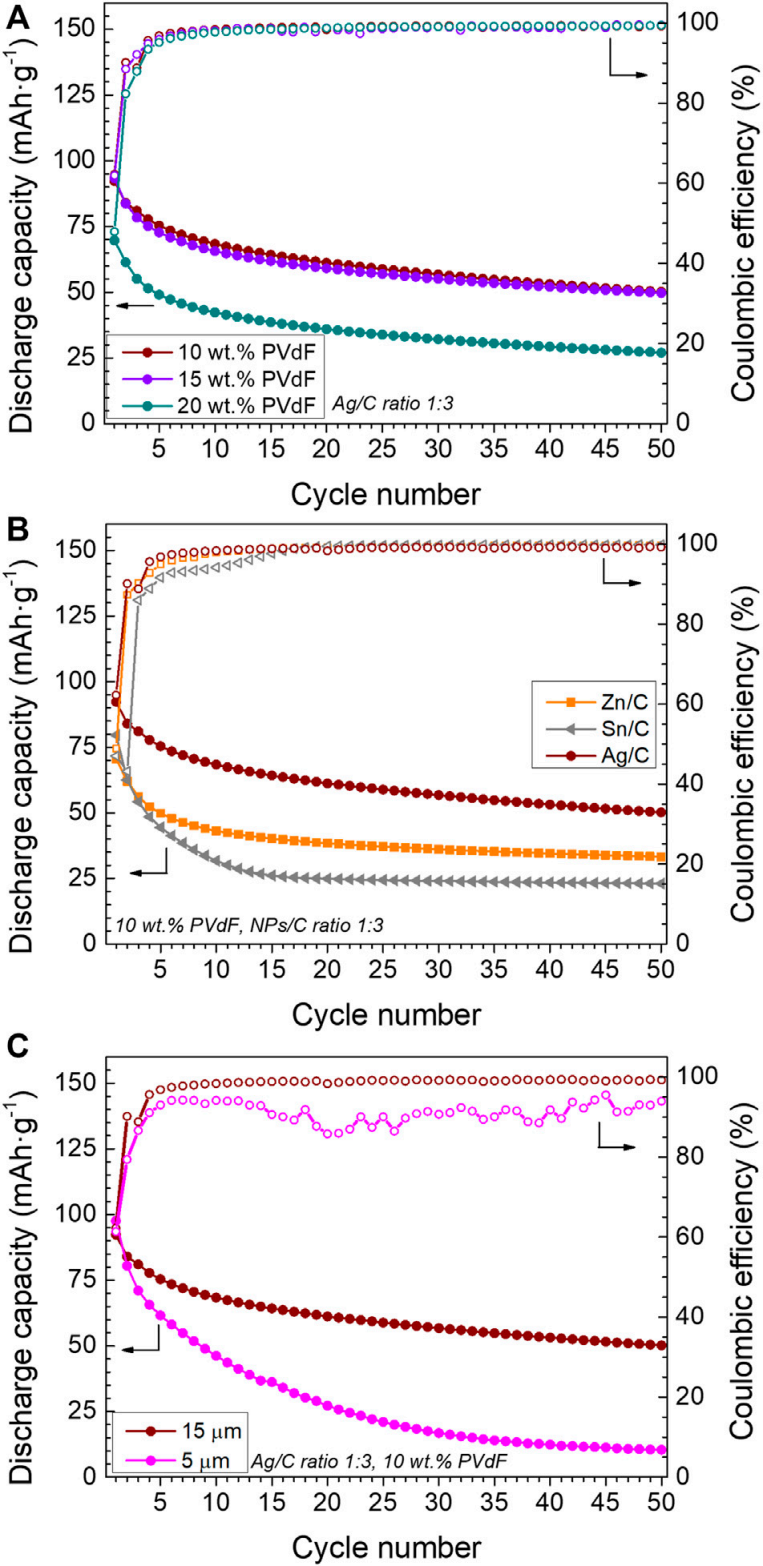
Discharge capacity and Coulombic efficiency of single-layer pouch cells using **(A)** Ag/C 1:3 ratio coatings with different PVdF content, **(B)** different metals in nanoparticles/C coatings, with 10 wt% PVdF, and NP/C ratio of 1:3, and **(C)** different thicknesses of Ag/C 1:3 ratio coatings with 10 wt% of PVdF. Cycling conditions: 60°C, 1 N m, DoD 100%, 2.5–3.8 V, 0.1C–0.1C, and charge cut off current 0.05C.

The amount of binder in the Ag/C nanocomposite layer has been varied to study its effect on the electrochemical behavior of anode free cells. Figure 3A shows the discharge capacity and the CE values of the investigated cells with CC coated by the Ag/C nanocomposite with different content of the PVdF binder. We can see an increase in the discharge capacity when maintaining all the experimental parameters and decreasing the amount of the PVdF binder from 20 down to 15 and 10 wt%, with similar behavior in these two cases. The discharge capacity of the first cycle increases from 70 up to 90 mAh·g^−1^, and a moderate capacity fading, with the same tendency, is maintained in the cells independently of the amount of the binder. Thus, the discharge capacity of the cell containing the coating with 10 wt% of PVdF in the layer is 50 mAh·g^−1^ with a capacity retention of 54% after 50 cycles, in comparison to a cell containing 20 wt% of PVdF, which exhibits 27 mAh·g^−1^, with a capacity retention of 39%. Although the electrochemical behavior is similar with 15 and 10 wt% of PVdF, there is a very slight improvement in discharge capacity and capacity retention using 10 wt% of PVdF. This effect is most probably caused by the decrease of the coating’s electronic conductivity when the amount of binder is higher, because PVdF binder is an excellent electronic insulator. The PVdF binder content lower than 10 wt% has been discarded to maintain the homogeneity and good mechanical properties of the coating layer.

Commercial nanoparticles of different metals have been used to make nanocomposite layers with 10 wt% of PVdF. Figure 3B shows the discharge capacity and the CE of cells containing such layers. The cell with the Ag/C nanocomposite layer exhibits a higher initial discharge capacity and better capacity retention in comparison to the cells containing other metal nanoparticles (Zn or Sn). In this sense, the cell with an Sn/C-based layer shows an initial discharge capacity higher than the Zn/C but an accentuated capacity loss with the cycle number. Concerning the CE, all tested cells exhibit an initial low value and tend to stabilize close to 100%, but in the case of cells with the Ag/C coatings, the initial CE is slightly higher and reaches stable values earlier.

After the selection of different parameters for an optimal layer composition, different thicknesses of such a composition have been deposited on the reference CC. The initial gap in the doctor blade, fixed at 200 µm, which leads to a layer thickness of 15 ± 1 µm, has been varied to analyze the impact on the electrochemical performance of the anode free cells. When the gap increases up to 350 µm, the obtained thicker layer on CC cracks after drying, which prevents its subsequent use in cells. When the gap is decreased down to 50 µm, the obtained thickness of the coating after drying is 5 ± 1 µm. Figure 3C compares the cycling of cells based on copper CC with different coating thicknesses. Cells with both coatings exhibit a similar discharge capacity for the first cycle, above 90 mAh·g^−1^ and a similar CE, above 60%. However, the cells containing the thinner coating layer presented a faster capacity fading, which led to a poor capacity retention of 11% after 50 cycles. In contrast, the cell containing the thicker coating retains 54% of the initial capacity. Moreover, the Coulombic efficiency of the cell containing the thicker coating is higher and more stable. This improvement could be related to the high volume of the host to accommodate the deposited lithium metal.

**FIGURE 4:**
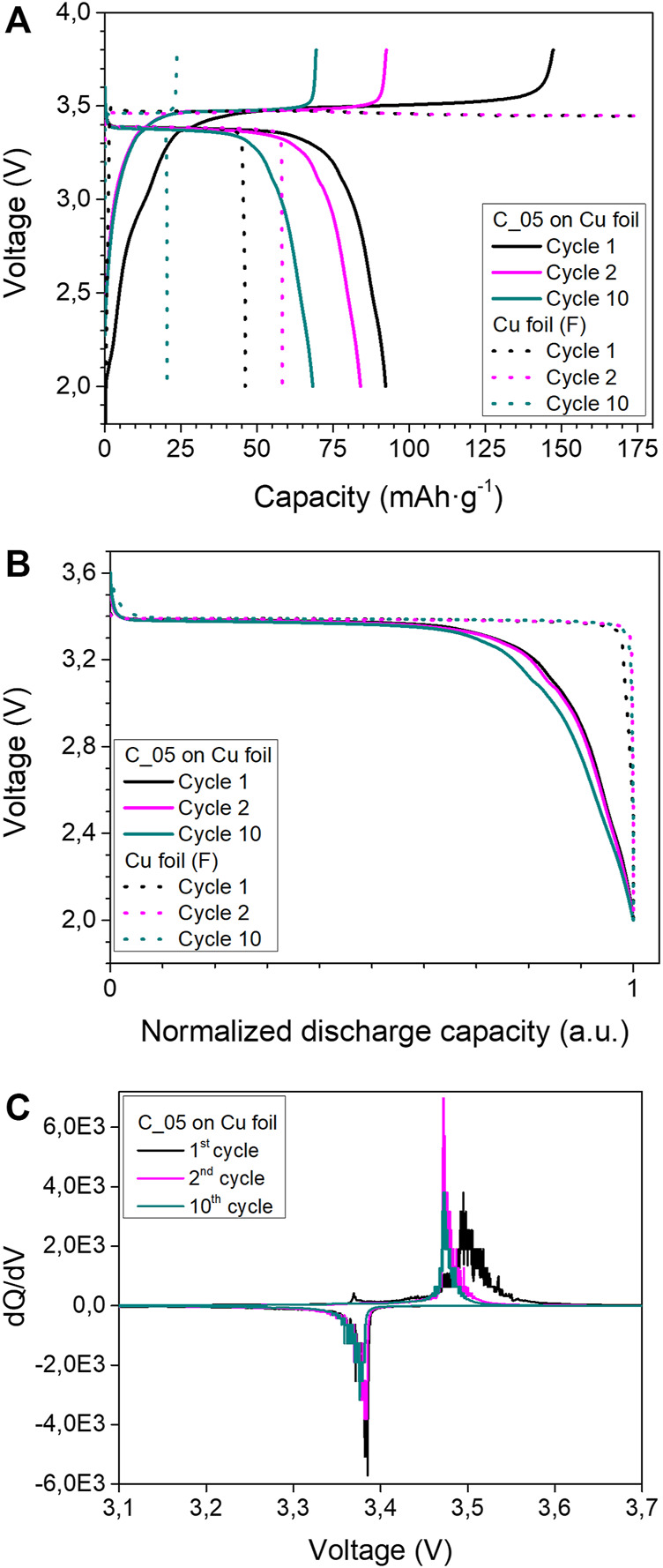
Electrochemical characterization of anode-free cells with the best coating (C_05) on Cu foil, and bare Cu foil as the anode current collector. **(A)** Charge/discharge profiles, **(B)** normalized discharge capacity of the first, second, and 10th cycles. **(C)** Differential capacity plots (dQ/dV) corresponding to the voltage profiles of the “C_05” based cell. Cycling conditions: 60°C, 1 N m, DoD 100%, 2.5–3.8 V, 0.1C–0.1C, and charge cut off current 0.05C.

Supplementary Table S1 summarizes all the coatings performed on the reference Cu foil anode current collector, together with the main electrochemical results of cells cycled containing such coatings. The anode-free solid-state cell, which contains a 15 µm thick nanocomposite layer of Ag/C with a 1:3 wt. ratio and a 10 wt% of the PVdF binder on the reference CC (coating C_05), has presented the best electrochemical results. The electrochemical performance of such anode-free cell, together with the cell using the bare Cu foil as anode, has been analyzed in detail. Figure 4A shows the charge–discharge profiles of both investigated cells for the first, second, and tenth cycle. The charge-discharge curves of both investigated cells are typical for Li/LiFePO_4_ electrochemical system. At the same time, the Cu foil based cell does not reach 3.8V during the first and second cycles, probably due to a nonuniform Li metal deposition which provokes cell micro-short-circuiting and results in low CE. In order to prevent irreversible damage and cell failure, the charging time was limited to 12 h. In turn, the cell based on the anode with the C_05 coating shows classical charge profiles, displaying much more efficient and uniform Li metal electrodeposition. Figure 4B shows the normalized discharge profiles of the investigated cells, demonstrating that the internal resistance of both anode-free cells is quite stable over cycling despite discharge capacity decay. Differentiated (dQ/dV) charge–discharge curves of the C_05-based cell presented in Figure 4C show a little shift of the charge peak after the first cycle and a reduction of the intensity of both charge and discharge peaks over cycling. Thus, such electrochemical behavior could be clearly attributed to the loss of cyclable lithium in the system that is typical for anode-free batteries. (Dubarry et al., 2014).

### 3.3 Post-Mortem Analysis

**FIGURE 5:**
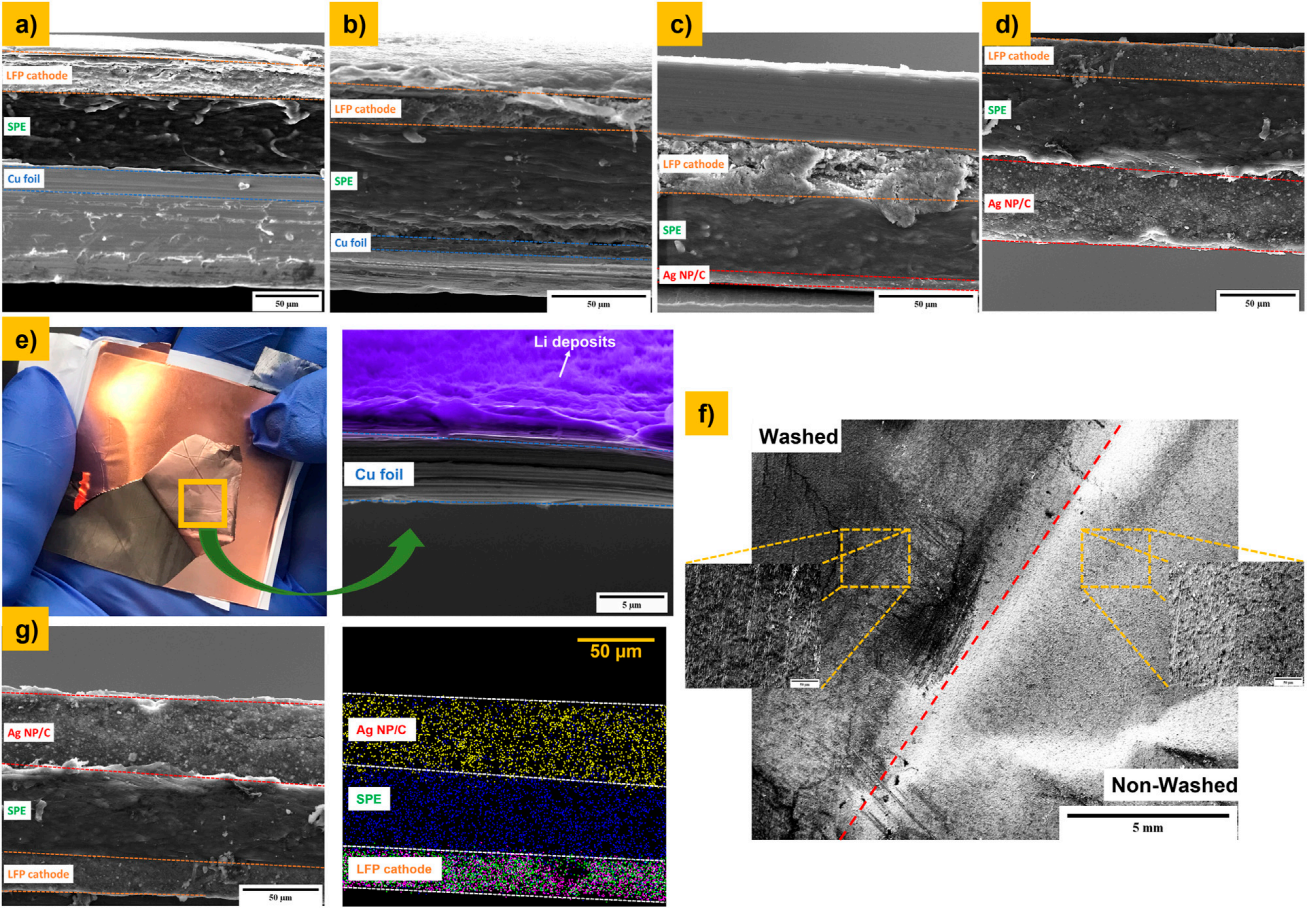
SEM Cross section of different pouch cells before and after charging processing up to 3.8 V. **(A)** AF_01, **(B)** AF_02, **(C)** AF_03, and **(D)** AF_04; **(E)** photograph taken during the disassembling of “AF_02” cell and the cross section of the Cu foil CC after charging the pouch cell up to 3.8 V; **(F)** top view SEM micrographs of Cu foil CC recovered from “AF_02” before and after the washing process; and **(G)** cross section and mapping of “AF_04” cell after the ion-milling process: Ag (yellow dots); S (blue dots); Fe (purple dots); and P (green dots).

Figures 5A–D shows the SEM micrographs of the cross-section of four investigated anode-free pouch cells (Table 3). In all micrographs, the presence of the different components of the pouch cell, such as the LFP cathode, solid polymer composite electrolyte, and the current collector, is evident. Regarding the pouch cells which have been charged at 3.8 V (“AF_02” and “AF_04”, Figures 5B,D), it is not so evident the deposition of Li, neither on Cu foil nor on the coating based on the Ag/C. Nevertheless, the cross section of the Cu foil recovered from the cell “AF_02” exhibits a rough surface and Li deposits on the surface of the current collector (see Figure 5E), whereas the Cu foil of the rest of the pouch cells shows a flat surface, indicating that the Li metal has not been plated. Indeed, during the disassembling of “AF_02” cell, a grayish/blackish surface on the inner side of Cu foil is observed, suggesting that Li metal can deposit on the surface of the current collector. In order to go deeper with this assumption, a small piece of Cu foil recovered from cell “AF_02” has been washed inside the glovebox, and the top view of the non-washed and washed part of the Cu foil has been analyzed by SEM (see Figure 5F). The non-washed area displays a rough surface, probably related to the deposition of Li metal on the current collector surface during charging, while the washed area exhibits a flatter and cleaner surface than its non-washed counterpart, evidencing the removal of plated Li after the washing process. On the other hand, Figure 5G shows the cross section and EDX elemental mapping of the pouch cell that has been assembled with the Ag/C coating as a current collector and charged at 3.8 V (“AF_04”). The different components of the cell are visible; however, it is not possible to observe the deposition of lithium metal along the coating based on Ag nanoparticles and carbon black. This finding indicates that in the case of the cell “AF_04” lithium metal is, probably, alloying with Ag during the charge, which significantly improves the electrochemical performance of the investigated anode-free solid-state cell. This result is in good agreement with the earlier publication (Lee et al., 2020). Nevertheless, additional in-depth investigation is required for a better understanding of the Li-Ag alloying mechanism towards further improvement of the reversibility of anode free cells with polymer-based electrolytes.

Although there are still several issues regarding the electrochemical performance of AFB, as it has been explained throughout this research study, the energy density of a solid-state anode-free cell has been projected to assess the potential future of this system. Based on a cell prototype which contains 20 stacks and the packaging, a LFP-based cathode with a loading of 2.0 mAh·cm^−2^, and a thin polymer-based solid electrolyte with a thickness of 30 µm, it is estimated that the replacement of the Li metal anode with a thickness of 50 µm by a coating of 15 µm on the same 8 µm Cu foil current collector increases the volumetric energy density by about 19%, with a slight reduction in gravimetric density (see Supplementary Figure S4). Future efforts are required to achieve the combination of such challenging values and align the development of this emerging technology with the required targets of the automotive industry.

## 4 Conclusions

In this work, different current collectors together with various surface modifications have been studied as negative electrodes in anode-free solid-state batteries with the LiFePO_4_ cathode and PEO-LiTFSI-based solid electrolyte, as a simpler, cost-effective, and more sustainable alternative to lithium metal and lithium-ion SSB.

Commercial copper foil has been selected as the reference anode current collector, and then coated with a composite layer containing metal nanoparticles, carbon black, and PVdF binder. The coating layer (C_05) composed of Ag nanoparticles and carbon black in a 1:3 wt. ratio has presented the most promising results among tested configurations. Afterwards, the PVdF binder content and coating thickness have been modified in such a way to obtain higher values of discharge capacity, Coulombic efficiency, and capacity retention during cycling.

With the best coating, the initial discharge capacity is doubled (from 46 to 93 mAh·g^−1^) with respect to the bare copper current collector, and the cells exhibit a Coulombic efficiency above 99% after 50 cycles, moderating the capacity fading after the first five cycles. The cells based on the “C_05” negative electrode have shown improved electrochemical performance, most probably due to enhanced homogeneity and reversibility of the Li-stripping/plating process, avoiding microshorting of the cell. However, the obtained discharge capacity of the best anode-free SSB configuration “C_05/LiFePO_4_” is still far from the accessible capacity of LiFePO_4_ material (e.g. 155 mAh·g^−1^) and the electrochemical performance of SSB employing Li metal anode. Thus, the investigated surface coating on the anode current collector slows down, although does not fully avoid, the loss of cyclable lithium and, as a result, capacity fades during battery cycling.

In this context, our future efforts will be concentrated on better understanding of the mechanism of Li-stripping/plating and interfacial phenomena to improve the reversibility of the whole anode-free battery. Additionally, we will investigate the effects of buffer layer formulation (type and particle size of nanoparticles, anolyte implementation, component ratio, and deposition of a thin Li metal seed layer, etc.), preparation methodology (mixing and coating homogeneity, etc.), and its geometry (thickness, density, porosity, etc.) on the electrochemical performance of anode-free solid-state batteries.

## Data Availability

The original contributions presented in the study are included in the article/Supplementary Material; further inquiries can be directed to the corresponding author.
