# Supplementary material for: Selection and Surface Modifications of Current Collectors for Anode-Free Polymer-Based Solid-State Batteries
Source: Front Chem. 2022 Jul 7;10:934365. doi: 10.3389/fchem.2022.934365 (PMC9300918; doi:10.3389/fchem.2022.934365)
Supplement: Supplementary file 1 [file DataSheet1.PDF]

Supplementary Material

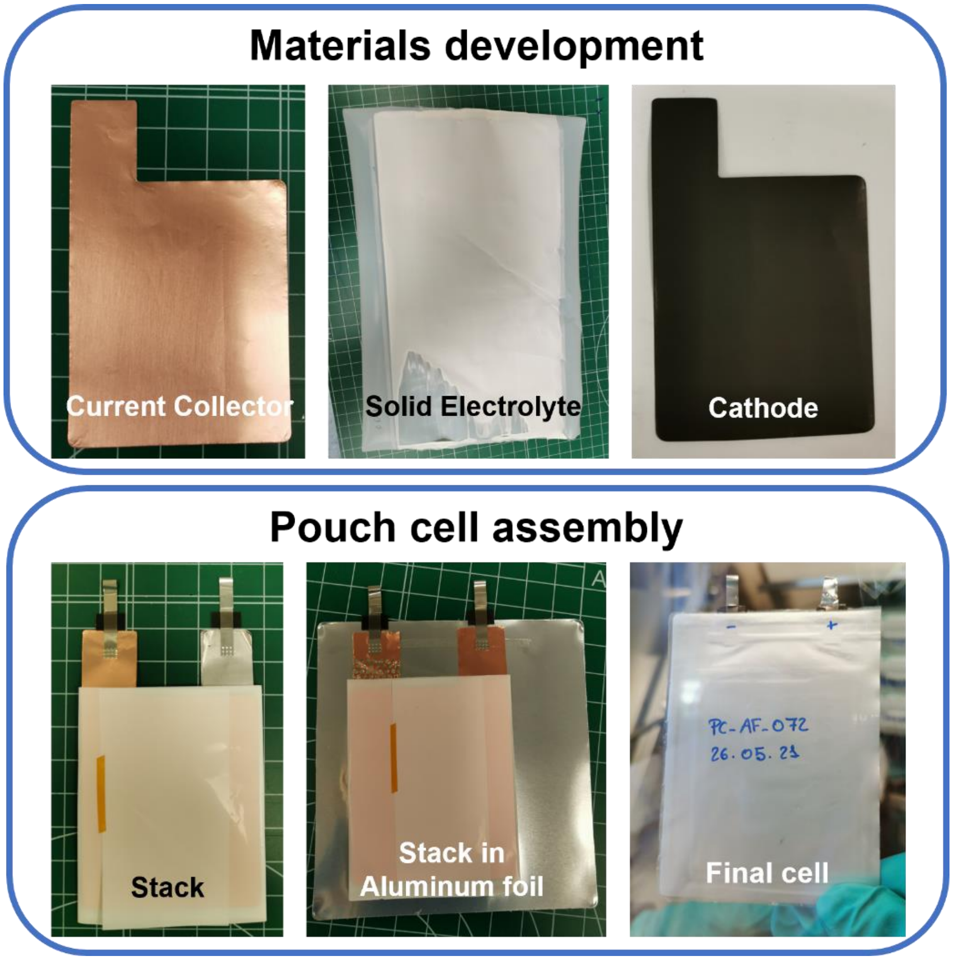

Supplementary Figure 1. Scheme of anode-free solid-state single-layer pouch cell assembly.

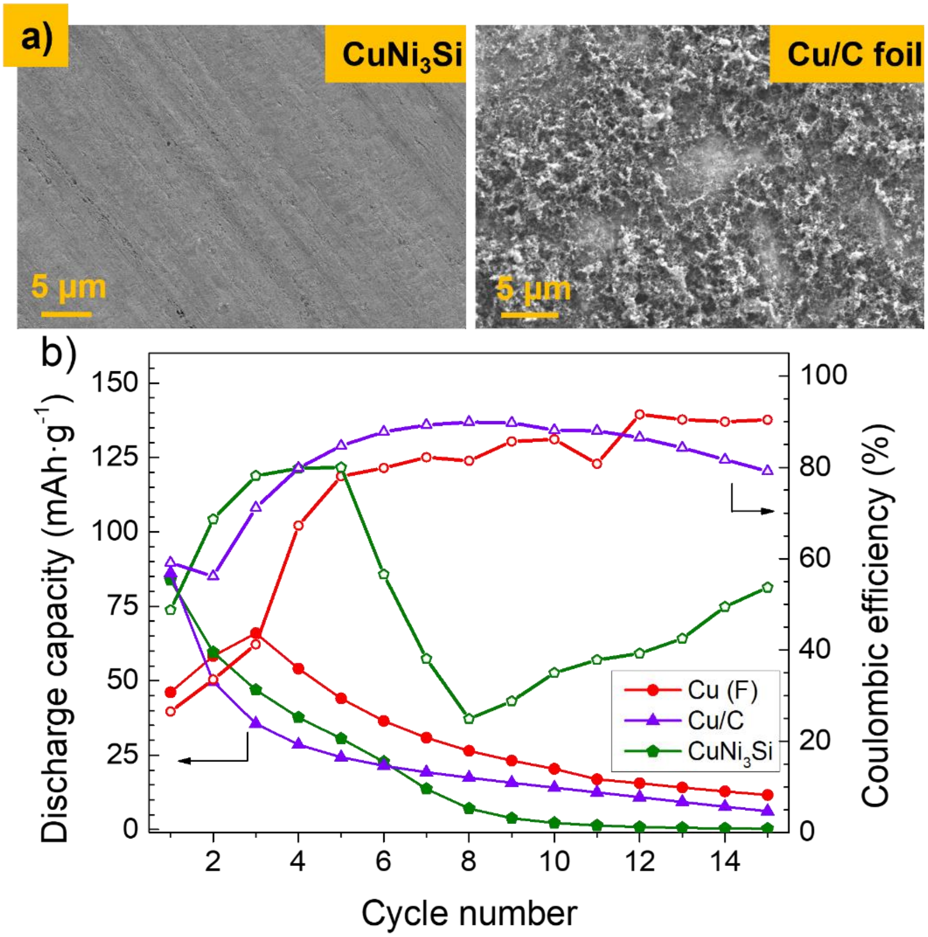

Supplementary Figure 2. (a) FE-SEM micrographs of alternative commercial materials studied as anode current collectors. (b) Discharge capacity and Coulombic efficiency of single layer pouch cells using such anode current collectors. Cycling conditions: 60 ºC, 1 N·m, DoD 100%, 2.5-3.8 V, 0.1C-0.1C, charge cut off current 0.05C.

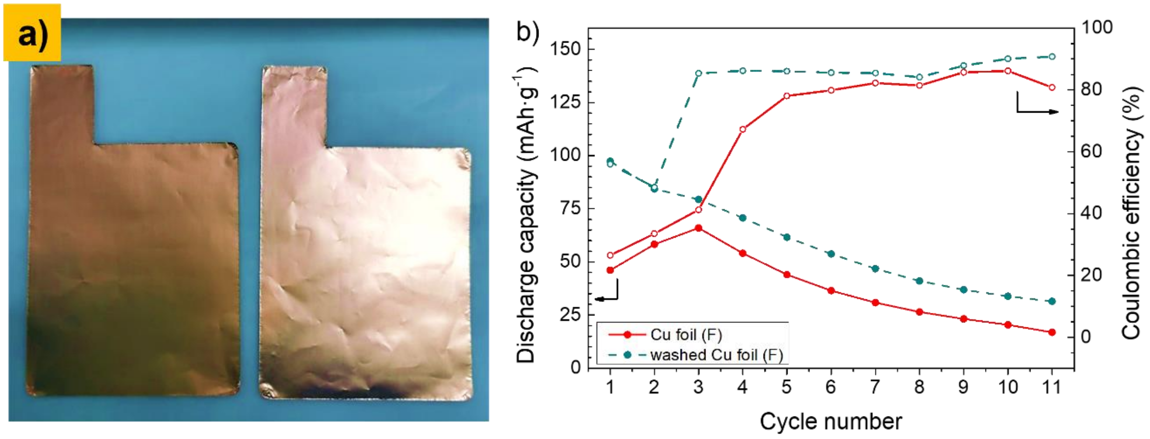

Supplementary Figure 3. (a) Digital photo of a Cu foil current collector (Furukawa), as received (left) and after chemical etching with HCl solution (right). (b) Cycling data comparison of cells using Cu foil as CC, as received and after HCl treatment. Cycling conditions: 60 ºC, 1 N·m, DoD 100%, 2.5-3.8 V, 0.1C-0.1C, charge cut off current 0.05C.

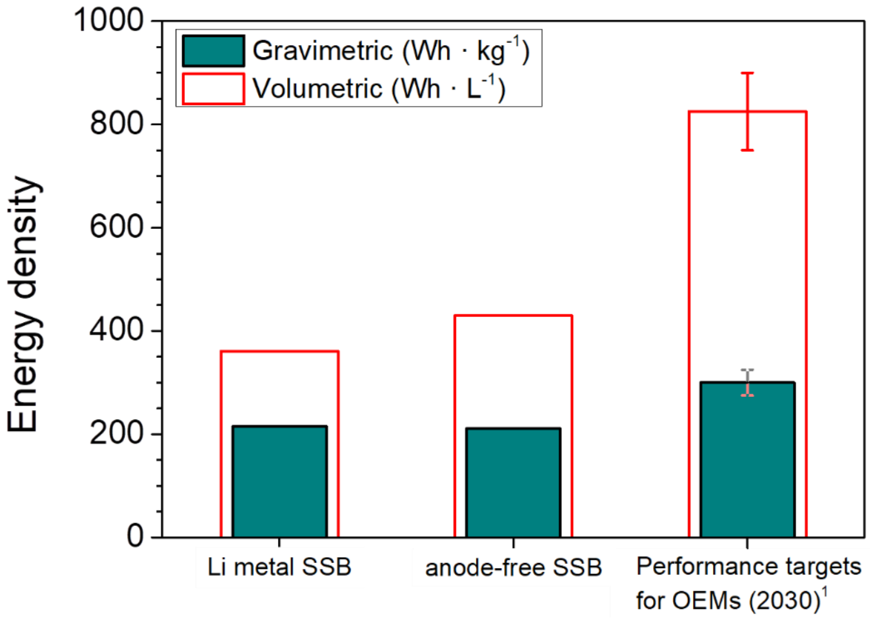

Supplementary Figure 4. Estimated gravimetric and volumetric energy density of an anode-free solid-state cell (containing 20 stacks, LFP cathode with a loading of 2.0 mAh·cm^{-2}, polymer-based solid electrolyte of 30 µm thickness), using C_05 coating on Cu foil current collector (total thickness 23 µm), and the analogous SSB using Li metal (50 µm) and Cu foil (8 µm) as anode. Comparison of the projected values with the targeted values defined by the OEMs for 2030.[1] (Note: estimation was made using a proprietary Excel tool).

1. Armand, M. *et al.* Lithium-ion batteries – Current state of the art and anticipated developments. *Journal of Power Sources* **479**, 228708 (2020).

Supplementary Table 1. List of coatings done on Cu foil as anode current collector, together with the electrochemical results of anode-free pouch cells containing these nanocomposite layers as anode active material.

**Coating on Cu foil anode current collector**				**1st cycle**		**50th cycle**		
**Name**	NPs	NPs:C ratio (wt.)	PVdF (wt.%)	Discharge capacity ($mAh\cdot g^{-1}$)	Coulombic efficiency (%)	Discharge capacity ($mAh\cdot g^{-1}$)	Coulombic efficiency (%)	Capacity retention (CR) (%)
**Bare Cu foil**	-	-	-	46.1	26.5	Stopped after 20 cycles (CR < 10%)		
**Washed Cu foil**	-	-	-	97.3	56.0	Stopped after 10 cycles (CR < 35%)		
**C_01**	Ag	1:3	20	69.7	47.9	27.1	99.4	39
**C_02**	Ag	1:2	20	82.5	56.1	17.8	98.0	22
**C_03**	Ag	1:1	20	70.3	48.3	19.4	99.4	28
**C_04**	Ag	1:3	15	93.3	62.0	49.7	99.4	53
**C_05 (15 µm)**	Ag	1:3	10	92.3	62.2	50.2	99.3	54
**C_06**	Zn	1:3	10	70.5	48.9	33.3	99.7	47
**C_07**	Sn	1:3	10	79.6	47.0	23.0	99.8	29
**C_05 (5 µm)**	Ag	1:3	10	97.6	61.4	10.4	94.0	11
**C_05 (25 µm)**	Ag	1:3	10	Cracked coating				
